# Cytomorphological spectrum of subcutaneous and intramuscular cysticercosis: A study of 22 cases

**DOI:** 10.4103/0970-9371.73294

**Published:** 2010-10

**Authors:** Meenu Gill, Shivani Dua, PS Gill, Veena Gupta, Sumiti Gupta, Rajiv Sen

**Affiliations:** Department of Pathology, Pt. B.D. Sharma Post Graduate University of Medical Sciences, Rohtak, Haryana, India

**Keywords:** Cysticercosis, fine needle aspiration cytology, parasite

## Abstract

**Background:**

Cysticercosis is more common than usually thought. Fine needle aspiration cytology (FNAC) plays an important role in prompt recognition of this disease.

**Aims:**

To study the role of FNAC in the diagnosis of cysticercosis.

**Materials and Methods:**

Twenty-two patients with subcutaneous and intramuscular nodules, who were clinically diagnosed as tuberculous lymphadenitis, reactive lymphadenitis, lipoma, neurofibroma and cysticercosis were included in the present study.

**Results:**

In nine cases, a definitive diagnosis of cysticercosis was obtained in the form of fragments of parasite bladder wall and, biopsy confirmed the diagnosis. In the rest 13 cases, larval fragments could not be identified on the aspirates and the diagnosis of parasitic inflammation was suggested on the basis of other cytomorphological findings. Follow-up biopsy confirmed the diagnosis of cysticercosis.

**Conclusions:**

FNAC in cysticercosis is a low-cost outpatient procedure. The cytological diagnosis is quite straightforward in cases where the actual parasite structure is identified in the smears. However, in other cases, presence of eosinophils, histiocytes which may be in palisaded clusters or not, a typical granular dirty background, etc., are the features which should always alert the pathologist to this possibility.

## Introduction

Human cysticercosis commonly manifests as subcutaneous and intramuscular nodules. It is endemic in America, Africa and Asia. In India, it is more common in northern parts. The common sites of involvement include brain, muscle, eye and heart. Man occasionally serving as the larval host of *Taenia solium* becomes infected either by drinking contaminated water or by eating uncooked vegetables infected with eggs.[1] The preoperative diagnosis of cysticercosis can be made by radio imaging [computed tomography (CT) scan and magnetic resonance imaging (MRI)] and serological tests like complement fixation test, hemagglutination, radioimmunoassay and enzyme linked immunosorbent assay (ELISA). Fine needle aspiration cytology (FNAC) is now available as a preoperative tool for the diagnosis of subcutaneous cysticercosis. The diagnosis is confirmed by the histopathological examination of the excised specimen. Apart from studying the role of FNAC in the diagnosis of subcutaneous cysticercosis, the present study was done to analyse the cytomorphology of actual parasite and to study the cytological features suggestive of cysticercosis in those cases where the actual parasite could not be demonstrated.

## Materials and Methods

FNAC is an outpatient procedure in our hospital. More than 8000 FNACs of all foci are performed every year. This study included 22 patients presenting with palpable subcutaneous and intramuscular nodules at different sites. FNAC was performed with 22-gauge needle. Aspirated material was smeared on the glass slides and stained with Papanicolaou stain after fixation in 95% alcohol and with May-Grünwald-Giemsa stain after air drying. Subsequent excision biopsy was also evaluated. The sections were reviewed and findings were correlated with the cytological findings.

## Results

The study included 22 patients in the age group 3–52 years. Nine patients in the present study were females and 13 were males. The mean age at diagnosis was 22.73 years. Eight cases presented with neck swelling, 7 with arm swelling, 3 with abdominal wall swelling, 2 with swelling axilla, one with swelling cheek and one with swelling breast. The clinical features of all patients are tabulated [Table 1]. All the patients presented with painless slow growing nodule, soft to firm in consistency, and the provisional diagnoses were lipoma, neurofibroma, tuberculous lymphadenopathy and cysticercosis. In seven cases, the aspiration yielded clear fluid and the rest of the cases yielded purulent fluid. All aspirations were performed without complications.

**Table 1:** Clinical and cytological features in 22 cases included in the present study

Case	Site	Clinical diagnosis	Cytology
1.	Left arm	Neurofibroma	Bladder wall of cysticercus, Ma
2.	Right neck	Tuberculous lymphadenitis	Bladder wall of cysticercus, N, Ma, Eo
3.	Right arm	Cysticercosis	Bladder wall of cysticercus
4.	Left neck	Tuberculous lymphadenitis	Bladder wall of cysticercus, Eo
5.	Left arm	Lipoma	Bladder wall of cysticercus
6.	Right cheek	Cysticercosis	Bladder wall of cysticercus, L
7.	Left neck	Cysticercosis	Bladder wall of cysticercus
8.	Right mandible	Cysticercosis	Bladder wall of cysticercus, L, epithelioid cells
9.	Abdominal wall	Cysticercosis	Bladder wall of cysticercus
10.	Right arm	Benign tumor	N, L, Ma
11.	Right axilla	Tuberculous lymphadenitis	Eo, N, L, Ma
12.	Right neck	Cysticercosis	N, Ma, epithelioid cell granulomas
13.	Abdominal wall	Benign tumor	N, L, Ma
14.	Right neck	Reactive lymphadenitis	N, Eo, H, epithelioid cell granulomas
15.	Left arm	Benign tumor	L, PC, Ma, Eo, epithelioid cell granulomas
16.	Abdominal wall	Benign tumor	N, L, Ma, PC, Eo, H
17.	Left arm	Lipoma	N, Ma, epithelioid cell granulomas
18.	Left breast	Benign tumor	Ma, N, calcified mass
19.	Right neck	Tuberculous lymphadenitis	Ma, N, L, acellular homogeneous wall-like material
20.	Right axilla	Lipoma	N, Ma, Eo
21.	Left neck	Reactive lymphadenitis	Eo, N, L, Ma, H
22.	Left arm	Cysticercosis, Hematoma	N, L, Ma, epithelioid cells

Ma–macrophages, N–neutrophils, L–lymphocytes, PC–plasma cells, H–histiocytes, Eo–eosinophils

**Figure 1:**
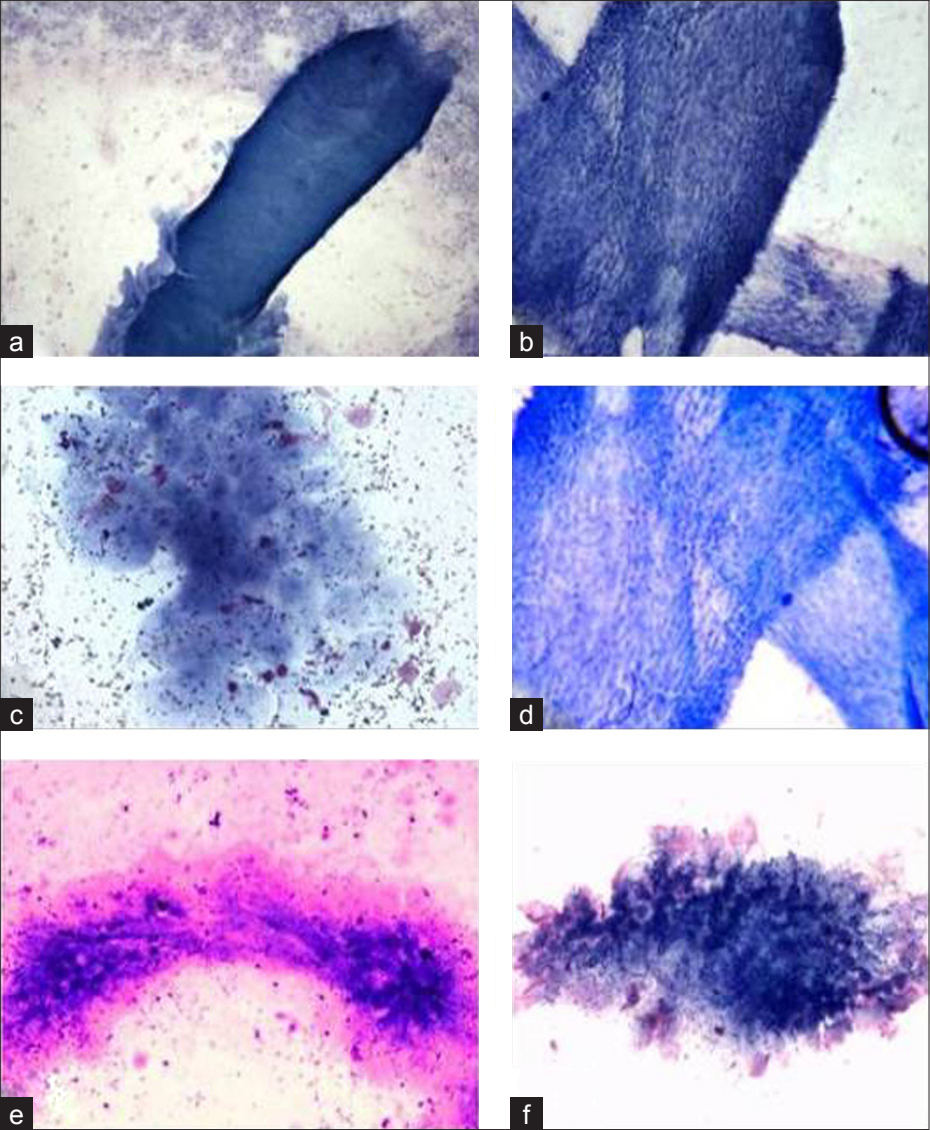
Photomicrograph showing wall of cysticercosis cellulosae (a and b) (Giemsa, ×100), (c–f) higher magnification (Giemsa, ×400)

**Figure 2:**
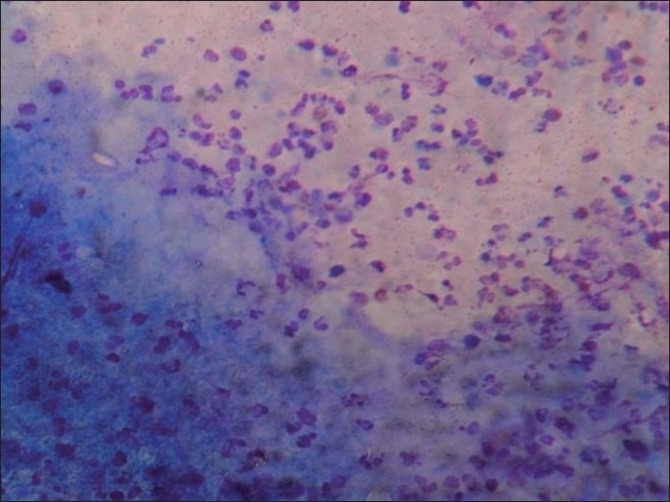
Wall of cysticercus with surrounding acute inflammatory cells (Giemsa, ×400)

**Figure 3:**
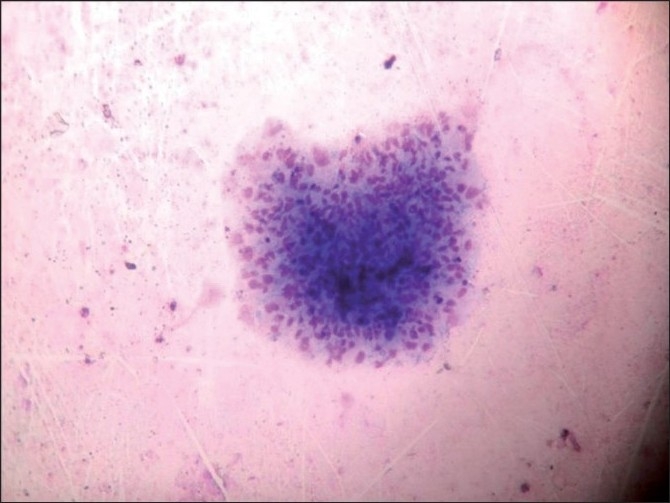
Photomicrograph showing epithelioid cell granulomas (Giemsa, ×400)

**Figure 4:**
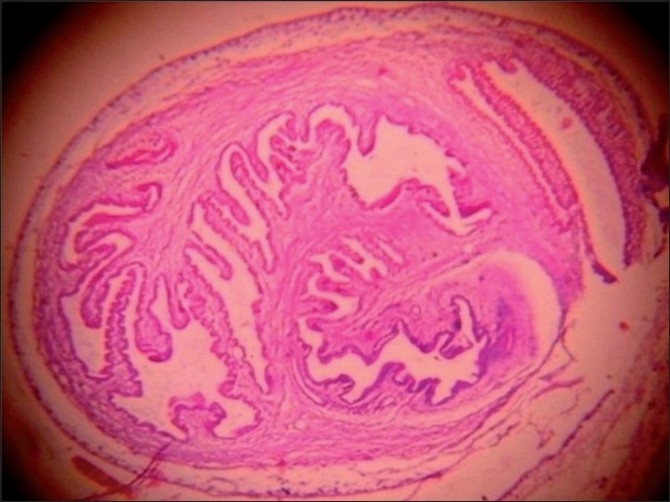
Photomicrograph of tissue section showing cysticercus larva enclosed in a thin fibrous cyst wall (H and E, ×100)

In nine cases on FNAC, actual parasite structures were demonstrable in the smears although the cytomorphology was not exactly the same in all cases [Figures 1–4]. None of these cases showed the presence of hooklets. Follow-up biopsy confirmed the diagnosis. In the rest 13 cases, the cytological findings were very much suggestive of a parasitic cyst; however, no parasite could be seen. The smears showed a mixed inflammatory infiltrate comprising neutrophils, lymphocytes, eosinophils, histiocytes and giant cells (in varying proportions in different cases). In 5 of these 13 cases, well-formed epithelioid cell granulomas were also evident. These cases were subjected to Ziehl Neelson staining which was noncontributory in all. No actual parasite structure could be demonstrated in any of these cases. A cytological diagnosis of parasitic cyst was suggested and excision was advised. Follow-up biopsy confirmed the diagnosis of cysticercosis.

## Discussion

Human cysticercosis is the larval infestation of the cestode *T. solium*. The cysticercus can be found in any organ, but is especially common in skeletal muscle, subcutaneous tissue, eyes and the central nervous system. Fully developed cysticerci are opalescent, milky white cysts, elongated to oval and about 1 cm in diameter. The cyst contains fluid and a single invaginated scolex. The scolex has a rostellum, four suckers and 22–32 small hooklets. The cyst wall is multilayered, 100–200 mm thick and covered by microvilli. The outer, cuticular layer appears smooth and hyalinized and is frequently raised in projections.[2] Beneath the tegument is a row of tegumental cells. The inner layer or parenchyma is loose and reticular, containing mesenchymal cells and calcerous corpuscles.[3] The calcareous corpuscles are a unique feature of cestode tissue. These spherical, noncellular masses occur in the parenchyma and are especially prominent in larval cestodes. The corpuscles take on a bluish purple color in hematoxylin and eosin (H and E).[4]

Cyticerci nodules in the skin are difficult to differentiate from benign mesenchymal tumors and lymphadenitis on clinical grounds alone.[5] The cytomorphological identification of larvae in FNAC smears by different workers has widened the diagnostic utility of FNAC in skin nodules.[2,5,6] Suspicion about a parasitic lesion starts with the presence of eosinophils, neutrophils, palisading histiocytes and giant cells in an aspirate from subcutaneous nodule. The diagnosis of cysticercus is made when fragments of larval cuticle and parenchyma are identified. The presence of scolex in cytology smears is an uncommon finding.[6–8] No scolex was seen in any of our aspirates.

Viable cysticerci may not cause any inflammatory response. However, when they degenerate, there is an infiltration of inflammatory cells, associated with the development of foreign body granulomas. The viable cyst and the necrotic and calcified lesions all have distinctive cytomorphological patterns. The viable cyst yields clear fluid and shows fragments of bladder wall in a clear acellular background. Aspirates of necrotic lesions may contain fragments of bladder wall, including calcareous corpuscles and detached single hooklets.[9]

FNAC in cysticercosis is a low-cost outpatient procedure. It is one of the tools for preoperative diagnosis and may even obviate the need for open biopsy.[10] The cytological diagnosis is quite straightforward in cases where actual parasite structure is identified in the smears. However, in other cases, the presence of eosinophils, histiocytes which may be in palisaded clusters or not, a typical granular dirty background, etc., are the features which should always alert the pathologist to this possibility. Nonetheless, in still some cases of cysticercosis, none of these features may be present, and the inflammatory infiltrate may also be variable. Cysticercosis is more common than usually thought. In all inflammatory/cystic/inflammatory cystic lesions, the possibility of cysticercosis should be kept in mind.
